# Supplementary material for: Paladin is a phosphoinositide phosphatase regulating endosomal VEGFR2 signalling and angiogenesis
Source: EMBO Rep. 2020 Dec 28;22(2):e50218. doi: 10.15252/embr.202050218 (PMC7857541; doi:10.15252/embr.202050218)
Supplement: Supplementary file 1 — Appendix [file EMBR-22-e50218-s001.pdf]

Paladin is a phosphoinositide phosphatase that regulates endosomal signaling and angiogenesis

Anja Nitzsche, Riikka Pietilä, Dominic T. Love, Chiara Testini, Takeshi Ninchoji, Ross O. Smith, Elisabet Ekvärn, Jimmy Larsson, Francis P. Roche, Isabel Egaña, Suvi Jauhiainen, Philipp Berger, Lena Claesson-Welsh and Mats Hellström

APPENDIX

S1: Control staining for Paladin after siRNA-mediated Paladin knockdown
S2: Actual p-values

Appendix figure S1

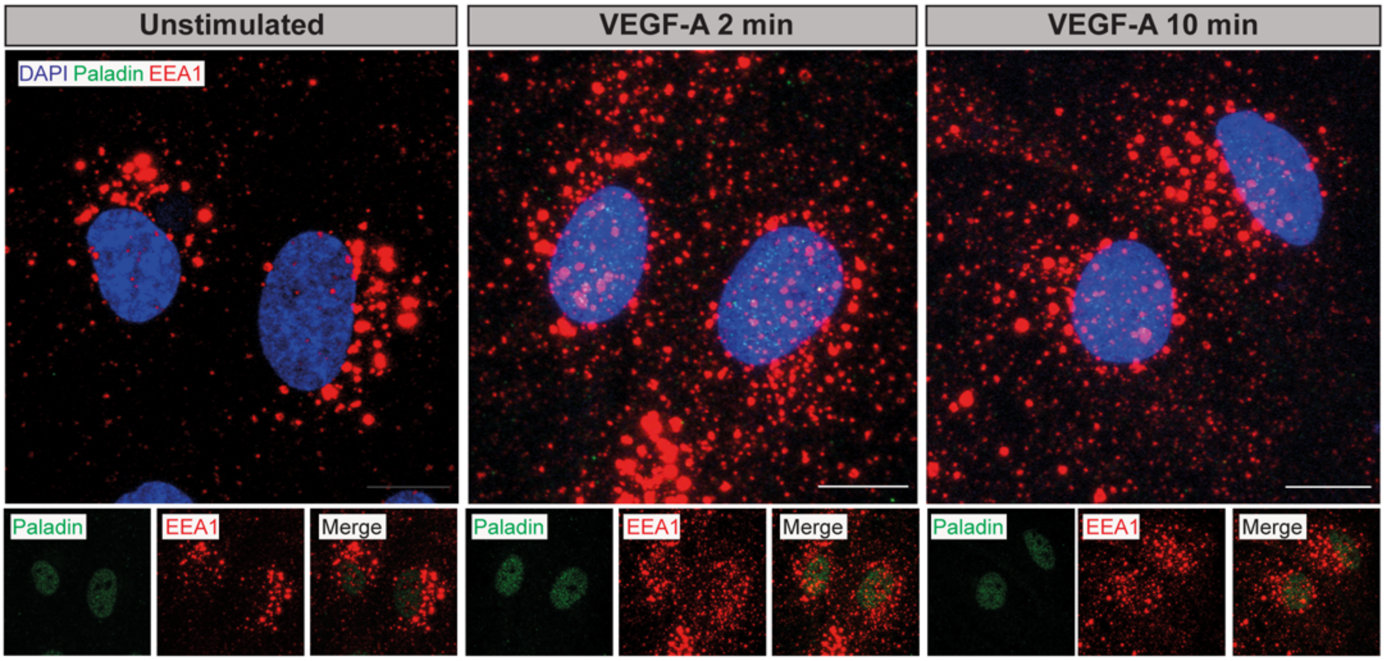

EEA1 (red), Paladin (green) and DAPI (blue) staining of siRNA *PALD1* knock-down cells after VEGF-A stimulation for indicated time points

Appendix S2

Exact p-values

Figure 1	Left	Right
a	0.0438	0.0362
d	0.0122	0.0040
f (top)	0.0009	<0.0001
f (bottom)	0.0062	0.0240
h	0.0387	

Figure 2	Left	Right
d	0.0136	
f (top)	0.0035	0.0364
f (bottom)	0.0021	0.0029
h (top)	<0.0001	
h (bottom)	0.0001	

Figure EV 2	Left	Right
a	0.0105	0.0006
d	0.0039	

Figure 3	Left	Right
b	0.0443	
d	0.0102	0.0035
f	0.0099	

Figure EV 3	Left	Right
a	0.0302	0.0017

Figure 4	Left	Right
b	0.0051	
d	0.0047	
f	0.0138	0.0004
h	0.0143	
i	0.0192	
l	0.0341	
m	0.0071	0.0151

Figure EV 4	Left	Right
a	0.0191	
b	0.0029	0.0403
c	0.0371 (first)	3.25E-10 (last)

Figure 5	
b	0.0265
e	0.0015
h	0.0451
