# Supplementary material for: Paladin is a phosphoinositide phosphatase regulating endosomal VEGFR2 signalling and angiogenesis
Source: EMBO Rep. 2020 Dec 28;22(2):e50218. doi: 10.15252/embr.202050218 (PMC7857541; doi:10.15252/embr.202050218)
Supplement: Supplementary file 2 — Expanded View Figures PDF [file EMBR-22-e50218-s002.pdf]

Expanded View Figures

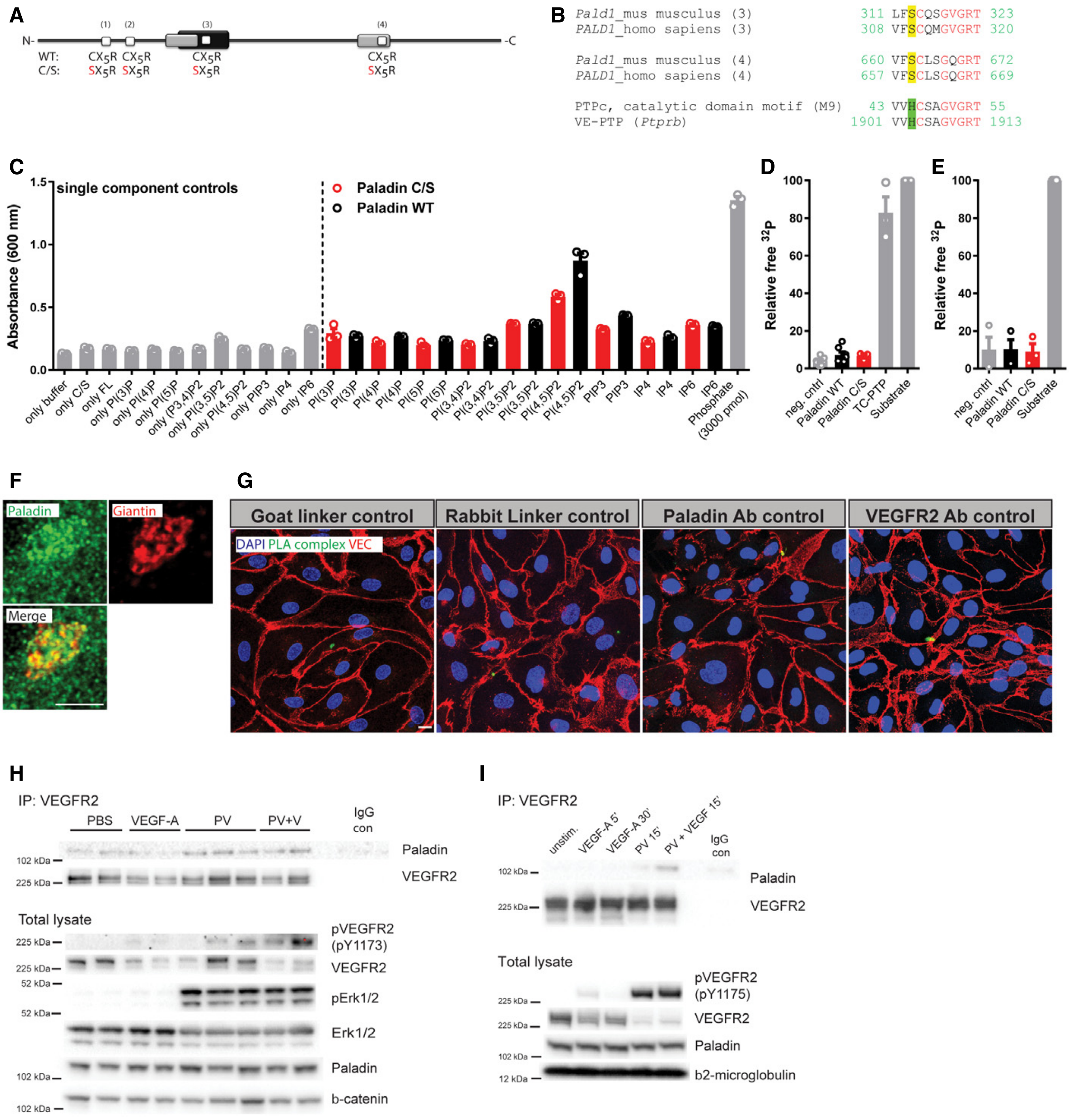

Figure EV1.

◀ **Figure EV1. Paladin is lipid phosphatase interacting with VEGFR2.**

A Schematic of Paladin protein depicting the four putative phosphatase domains (white boxes, CX_5R; X: any amino acid; mouse: amino acids 121–127, 160–166, 315–321, and 664–670; human: amino acids 118–124, 157–163, 312–318, and 661–667). Phosphatase domains predicted by Interpro (black box) and SCOP SUPERFAMILY algorithm (grey). A full-length phosphatase-dead variant was generated by substituting the four cysteine residues for serine (C/S).

B Amino acid sequence alignment of the third (amino acids: mouse, 312-–322; human, 309–319) and fourth (amino acids: mouse, 661–671; human, 658–668) putative phosphatase domains of Paladin with the consensus sequence of catalytic domain motif 9 of cysteine-based protein tyrosine phosphatases revealed serine instead of histidine residue in front of the cysteine in the phosphatase domains. By contrast, the phosphatase domain of vascular endothelial PTP (VE-PTP, *Ptprb*) contains the complete PTP motif.

C Screening of the phosphatase activity towards phosphoinositides and inositol phosphates (IP_4 = Ins(1,3,4,5)P_4 and IP_6 = Ins(1,2,3,4,5,6)P_6 of immunoprecipitated wild-type Paladin and phosphatase-dead (C/S) mutant variant using an *in vitro* colorimetric molybdate dye assay. Commercial SHIP2 enzyme reaction buffer was used (Echelon, USA). Mean ± SEM, n = 3 technical replicates.

D *In vitro* radioactive phosphatase assay using Paladin, wild-type, or C/S variant, immunoprecipitated from HEK293, and as a substrate, Src-optimized peptide phosphorylated on tyrosines. Immunoprecipitates from cells transfected with empty vector or endogenous TC-PTP served as negative and positive controls, respectively. Data were normalized to ^{32}P input. Mean ± SEM. n = 7 for wild-type Paladin, negative control, and substrate, n = 3 for C/S Paladin variant and TC-PTP (biological replicates).

E Immunoprecipitated full-length wild-type Paladin or its phosphatase-dead C/S variant expressed in HEK293 cells were analysed in an *in vitro* radioactive phosphatase assay using phosphorylated PKC-optimal peptide containing phosphoserine and phosphothreonine residues as a substrate. Immunoprecipitates of cells transfected with an empty vector served as a negative control. Data were normalized to ^{32}P input. Mean ± SEM. n = 3 biological replicates.

F Confocal representative image of HDMEC stained for Paladin (green) and Giantin (Golgi apparatus marker) (red). Cells were treated with VEGF-A (50 ng/ml) for 10 min. Scale bar: 5 μm.

G Controls for proximity ligation assay (PLA) to determine complex formation between Paladin and VEGFR2 as presented in Fig 1E. The indicated component was omitted in the reaction. HDMEC were counterstained for VE cadherin (red) and nuclei (DAPI, blue). Scale bar: 10 μm.

H Formation of VEGFR2/Paladin complex *in vivo*. Immunoprecipitation (IP) of VEGFR2 from lysate of wild-type adult mouse lung retrieved 2 min after tail vein-injection of VEGF-A (0.25 μg/g body weight) and/or peroxyvanadate (PV) (50 μmol/g body weight) or PBS, and immunoblotting for Paladin and VEGFR2. IP with isotype control IgG as negative control (IgG con). Each lane represents lysate from one mouse lung. Total lysate samples indicate levels of total and phosphorylated VEGFR2 and Erk and Paladin after above treatment. Beta-catenin serves as loading control.

I Formation of VEGFR2/Paladin complex *in vitro*. Immunoprecipitation (IP) of VEGFR2 from untransfected HDMEC stimulated with 50 ng/ml VEGF-A alone or in combination with phosphatase inhibitor 100 μM peroxyvanadate (PV) for indicated time points, and immunoblotting for Paladin and VEGFR2. IP with isotype control IgG as negative control (IgG con). Total lysate samples indicate level of total and phosphorylated VEGFR2 and Paladin after above treatment. Beta2-microglobulin serves as loading control.

Source data are available online for this figure.

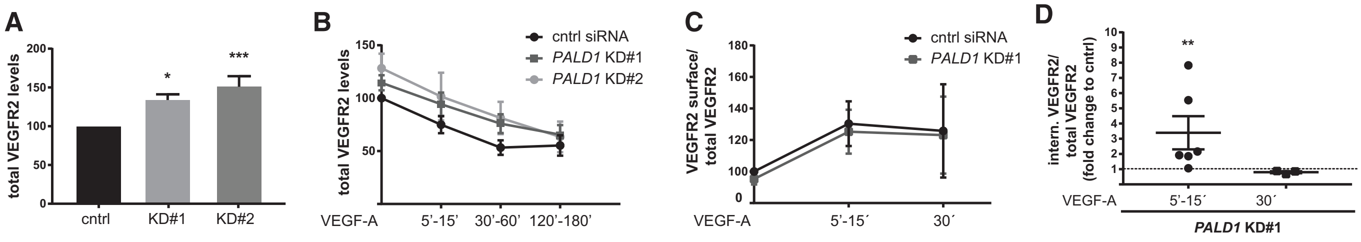

Figure EV2. Paladin regulates total and internalized VEGFR2 levels after VEGF-A stimulation of HDMEC.

A Total VEGFR2 was quantified from Western blots (Fig 2A and C) in HDMEC treated with control siRNA (cntrl) or siRNA targeting *PALD1* (KD#1 or KD#2) for 72 h. Mean ± SEM, one-way ANOVA. n = 13 biological replicates.

B Total VEGFR2 was quantified in HDMEC treated with control siRNA or siRNA targeting *PALD1* (KD#1 or KD#2), as shown in Fig 2A. Time-dependent degradation was observed after VEGF-A stimulation in both control and *PALD1* siRNA-treated cells. n = 4 biological replicates, Mean ± SEM.

C Quantification of VEGFR2 surface levels in *PALD1* #KD1 cells, as shown in Fig 2C. VEGFR2 surface levels (data pooled for the indicated time points) were normalized to total VEGFR2 levels in the lysate and compared between control and siRNA-treated HDMEC. Mean ± SEM. n = 4 biological replicates.

D Quantitative analysis of internalized VEGFR2, data shown for *PALD1*#KD1, as depicted in Fig 2D. Data were normalized to total VEGFR2 levels in the lysate after subtraction of signals in biotinylated and stripped samples. Mean ± SEM, t-test for indicated time points, normalized to cntrl siRNA sample. n = 3 biological replicates.

Data information: *P < 0.05, **P < 0.01, ***P < 0.001.

 © 2020 The Authors

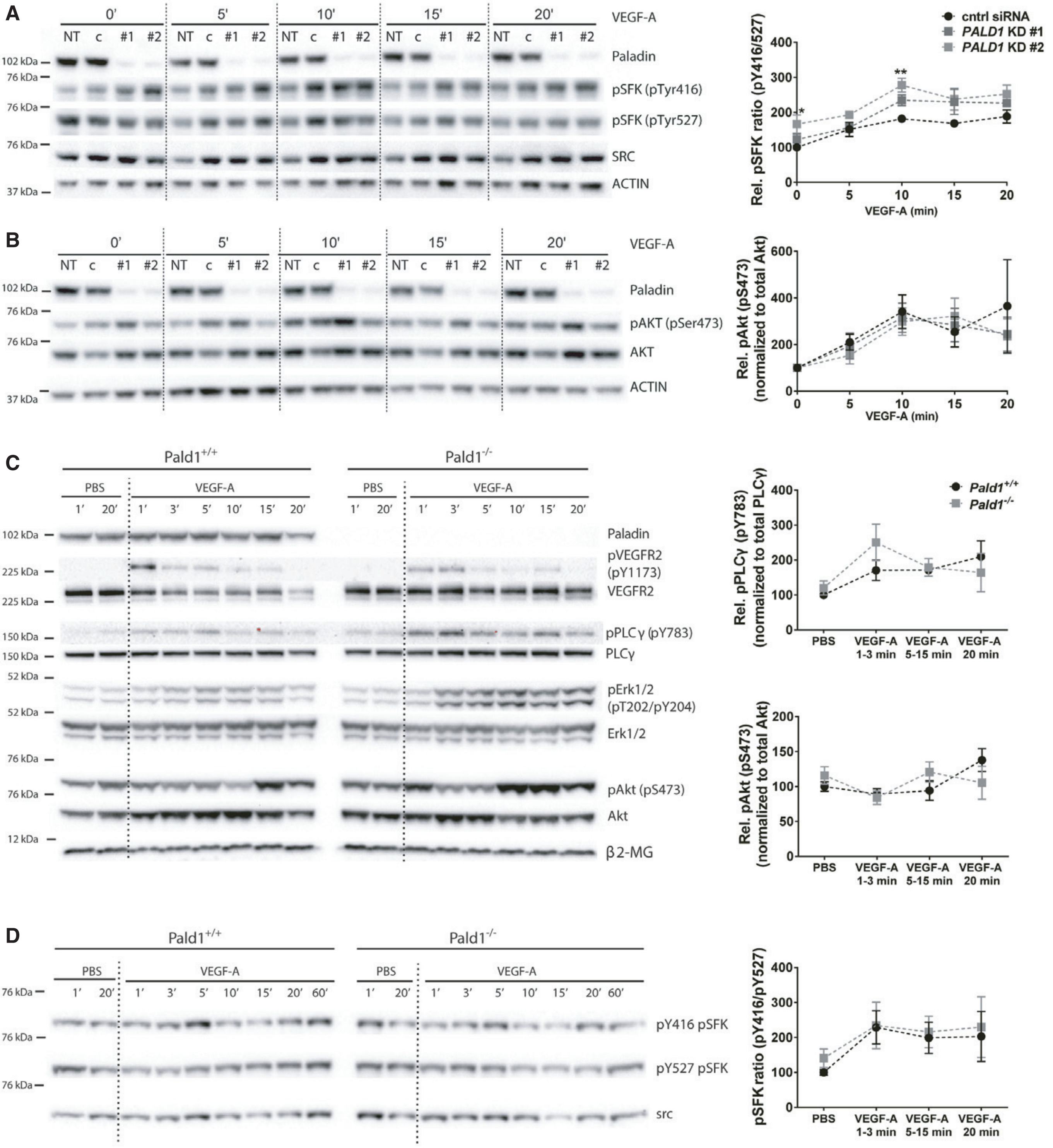
A
0' 5' 10' 15' 20' VEGF-A
NT c #1 #2
Paladin
pSFK (pTyr416)
pSFK (pTyr527)
SRC
ACTIN
cntrl siRNA
PALD1 KD #1
PALD1 KD #2
Rel. pSFK ratio (pY416/527)
VEGF-A (min)
B
Paladin
pAKT (pSer473)
AKT
ACTIN
Rel. pAkt (pS473) (normalized to total Akt)
VEGF-A (min)
C
Pald1+/+
Pald1-/-
PBS
VEGF-A
Paladin
pVEGFR2 (pY1173)
VEGFR2
pPLCγ (pY783)
PLCγ
pErk1/2 (pT202/pY204)
Erk1/2
pAkt (pS473)
Akt
β2-MG
Rel. pPLCγ (pY783) (normalized to total PLCγ)
Rel. pAkt (pS473) (normalized to total Akt)
PBS
VEGF-A 1-3 min
VEGF-A 5-15 min
VEGF-A 20 min
D
Pald1+/+
Pald1-/-
pY416 pSFK
pY527 pSFK
src
pSFK ratio (pY416/pY527)

Figure EV3.

Figure EV3. VEGF-A/VEGFR2 downstream signalling *in vitro* and *in vivo*.

A, B HDMEC, non-transfected (NT) or transfected with non-targeting (c/cntrl) or *PALD1*-targeting siRNA (KD #1, KD #2), were treated with VEGF-A for the indicated time periods. Immunoblots of cell lysates to determine protein levels of ACTIN, Paladin, pTyr416, and pTyr527 of Src family kinases (SFK) and total Src (A), pSer473 AKT and total AKT (B), are shown (left) and their quantification (right). Mean ± SEM, two-way ANOVA, $n = 3$ biological replicates.

C Immunoblotting of total heart lysates from $Pald1^{+/+}$ and $Pald1^{-/-}$ mice, tail vein injected with VEGF-A or PBS for the indicated time points, for Paladin, phosphorylated and total levels of VEGFR2, phospholipase Cγ (PLCγ), Erk1/2, and Akt, and β2-microglobulin (β2-MG, loading control). Part of the same blot is also shown in Fig 3. Quantification of pY783 PLCγ normalized to PLCγ ($n = 4$ biological replicates) and pS473 Akt normalized to Akt ($n = 5$ biological replicates), Mean ± SEM, two-way ANOVA.[§§]

D $Pald1^{+/+}$ and $Pald1^{-/-}$ mice were tail vein injected with VEGF-A or PBS for the indicated time periods. Heart lysates were blotted to determine the phosphorylated and total levels of Src family kinases (SFK). pY416 SFK normalized to pY527 SFK. Mean ± SEM, two-way ANOVA, $n = 5$ biological replicates.

Data information: *$P < 0.05$, **$P < 0.01$.

Source data are available online for this figure.

Figure EV4. Loss of *Pald1* leads to hypersprouting of the retinal vasculature.

A Quantification of the relative number of Erg positive endothelial cell (EC) nuclei around veins per field in *Pald1* knockout when normalized wild type. $n = 6$ per genotype, biological replicates. Mean ± SEM, Unpaired *t*-test.

B Quantification of spouting from endothelial spheroids in 3D collagen/methylcellulose gels. Spheroids of HUVEC untransfected or transfected with siRNA (control or *PALD1* #1 and #2) were grown with or without VEGF-A 50 ng/ml for 24 h and cumulative sprout lengths was quantified. Spheroids from HUVEC transfected with control siRNA without VEGF-A was set to 100%. Mean ± SEM, $n = 6$ ($n = 3$ for untransfected controls; one statistical outlier removed for *PALD1* siRNA without VEGF), one-way ANOVA.

C Quantification (left) of HDMEC transfected with negative control or *PALD1* siRNA (#2) grown under basal conditions and monitored for proliferation using Incucyte for 72 h. Mean ± SEM, two-way ANOVA, followed by Sidak's multiple comparison test. $n = 3$ biological replicates. Microscope images (right) showing confluency (number in per cent) at indicated time points. Scale bar: 300 μm.

D Representative images of Cyclin D1 (red) immunostaining in P5 retina showing nuclear localization in retinal vessels (isolectinB4, white). Scale bar: 50 μm.

E Western blot analysis of pT202/pY204 Erk1/2 levels in the P5 retina following a single intraperitoneal injection of MEK inhibitor U0126 (5 mg/kg), analysed after the indicated time periods and overnight (o/n).

F Representative images from stereomicroscope of retinas stained for isolectinB4 (white) from $Pald1^{+/+}$ and $Pald1^{-/-}$ P5 pups treated with vehicle or MEK inhibitor (U0126). Quantification in Fig 4L. Red arrow indicates distance from centre to the periphery of the vasculature in the vehicle-treated wild type. Scale bar: 500 μm.

G Representative confocal z-stacks of retinas stained for isolectinB4 (white) from $Pald1^{+/+}$ and $Pald1^{-/-}$ P5 pups treated with vehicle or MEK inhibitor (U0126). Quantification in Fig 4M. Scale bar: 100 μm.

Data information: *$P < 0.05$, **$P < 0.01$, ***$P < 0.001$, ****$P < 0.0001$.

Source data are available online for this figure.

[§§]Correction added on 3 February 2021, after first online publication: pPLCg has been changed to pPLCγ in the Y-axis title in Fig EV3C.

 © 2020 The Authors

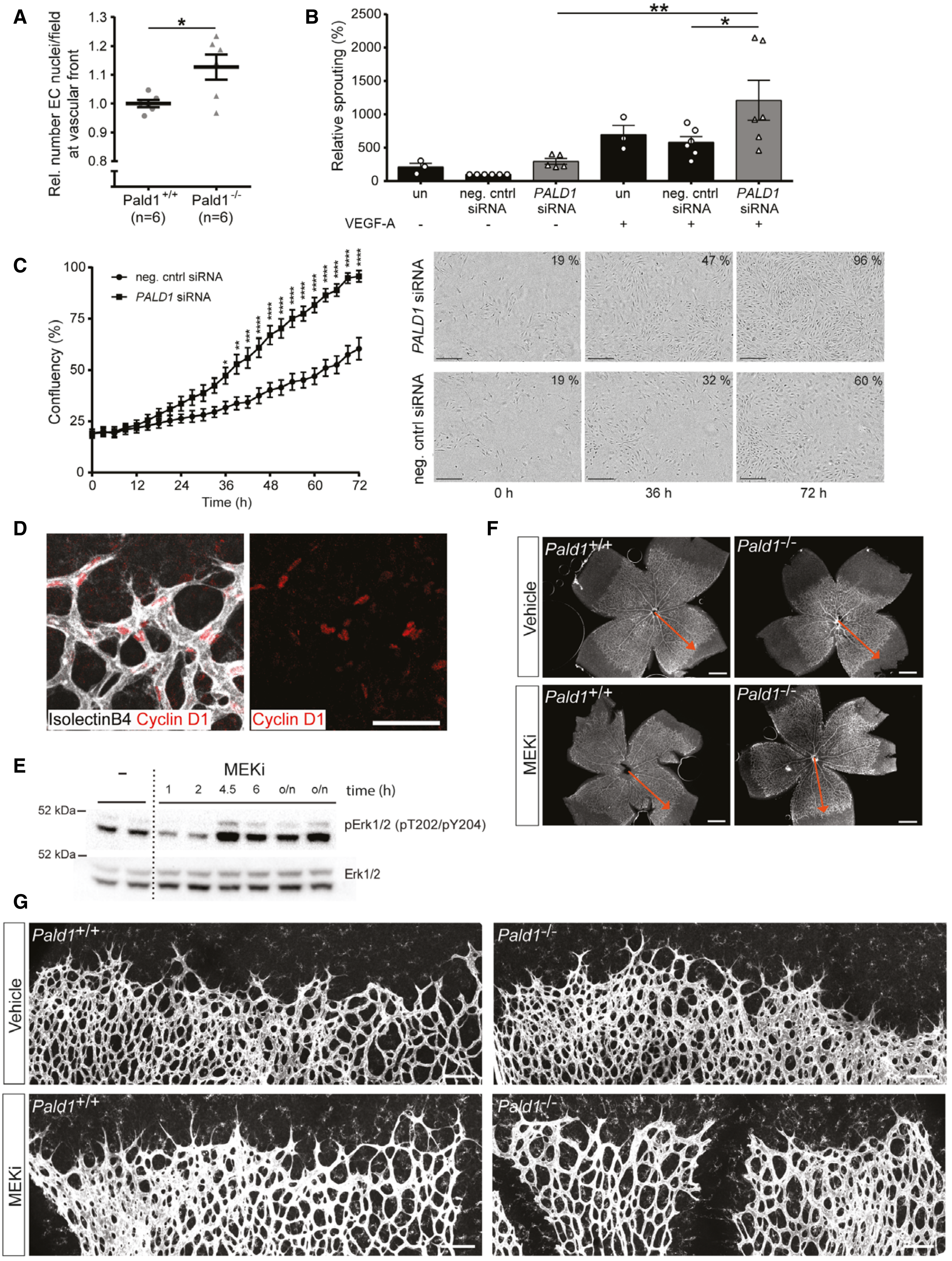
A
Rel. number EC nuclei/field at vascular front
1.3
1.2
1.1
1.0
0.9
0.8
*
Pald1 +/+ (n=6)
Pald1 -/- (n=6)
B
Relative sprouting (%)
2500
2000
1500
1000
500
0
**
*
un
neg. cntrl siRNA
PALD1 siRNA
VEGF-A
C
Confluency (%)
Time (h)
neg. cntrl siRNA
PALD1 siRNA
19 %
47 %
96 %
32 %
60 %
0 h
36 h
72 h
D
IsolectinB4 Cyclin D1
Cyclin D1
E
MEKi
time (h)
1
2
4.5
6
o/n
52 kDa
pErk1/2 (pT202/pY204)
Erk1/2
F
Vehicle
MEKi
Pald1+/+
Pald1-/-
G
Vehicle
MEKi
Pald1+/+
Pald1-/-

Figure EV4.

Figure EV5. Paladin does not affect leakage or VE-cadherin phosphorylation in pathological retinal angiogenesis.

A Immunoblotting for PALADIN in HUVEC, untreated, or treated for 24 h with VEGF-A (50 ng/ml), FGF2 (50 ng/ml), or SDF1α (30 ng/ml), actin served as loading control. Quantification in Fig 5B.

B Representative images of isolectinB4 stained retina of P15 mouse during OIR development, showing similar levels of vessel dropout between the genotypes caused by hyperoxic conditions at P7–P12. Scale bar: 500 μm.

C Representative images of microsphere extravasation from neovascular tufts indicated by isolectinB4 staining (red) following intravenous injection of 25 nm fluorescent microspheres (green) into mice that had been subjected to OIR. White arrows emphasize the extravascular accumulation of microspheres. Scale bar: 25 μm.

D Quantification of microsphere extravasation in the *Pald1*$^{+/+}$ and *Pald1*$^{-/-}$ retinas at P17 during OIR, as shown in (C). Mean ± SEM. Unpaired *t*-test, $n = 3$ litters, 4 pups per genotype.

E Representative images of VE cadherin (green), pY685 VE cadherin (red), and isolectinB4 (cyan) in the P17 retina of mice subjected to OIR. Scale bar: 50 μm.

F Quantification of pY685 VE cadherin in the *Pald1*$^{+/+}$ and *Pald1*$^{-/-}$ retina at P17 during OIR, as shown in (E). Mean ± SEM, Unpaired *t*-test, 5–7 pups per genotype.

Source data are available online for this figure.

© 2020 The Authors

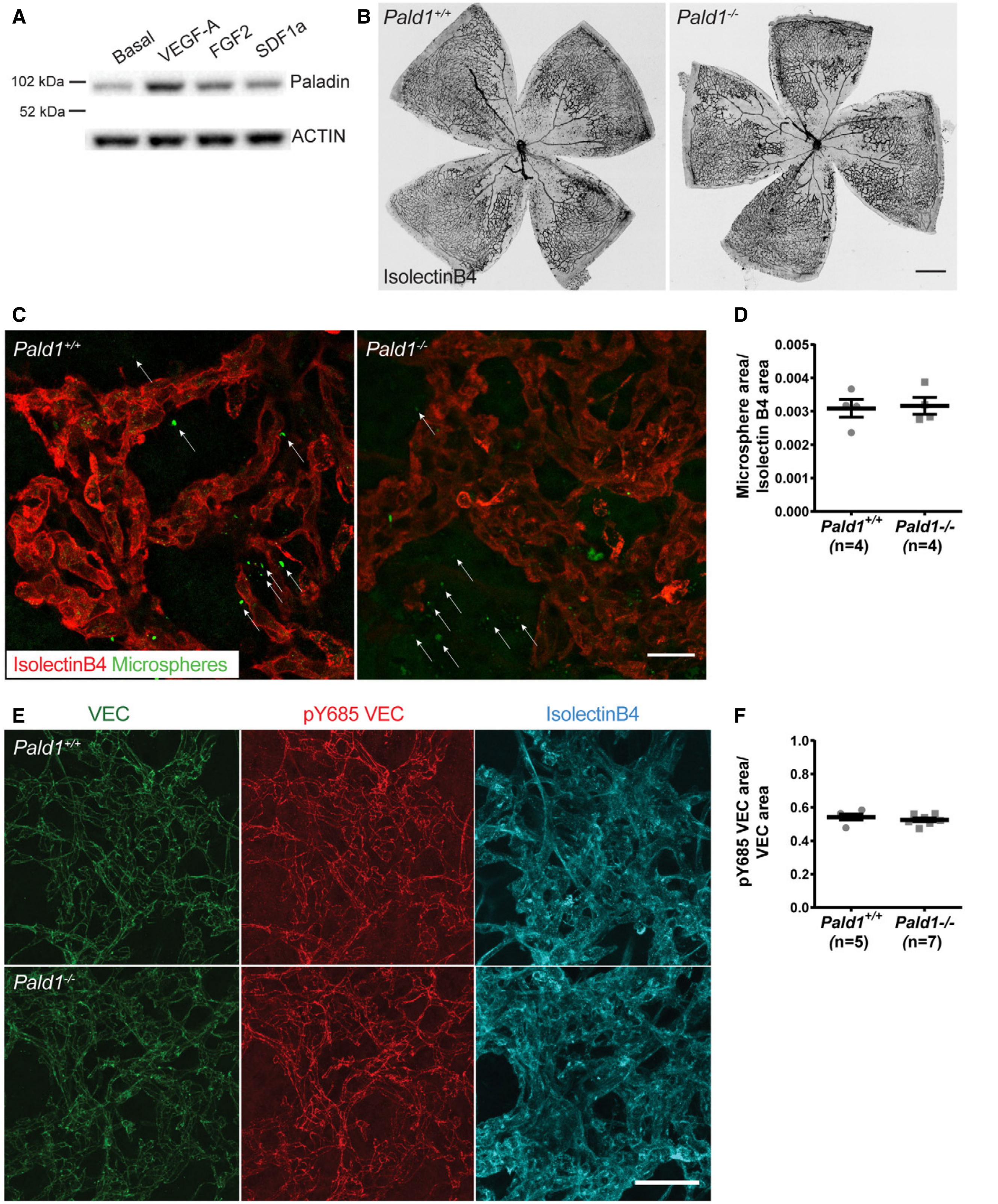
A
Basal
VEGF-A
FGF2
SDF1a
102 kDa
Paladin
52 kDa
ACTIN
B
Pald1+/+
Pald1-/-
IsolectinB4
C
Pald1+/+
Pald1-/-
IsolectinB4 Microspheres
D
Microsphere area/ Isolectin B4 area
0.005
0.004
0.003
0.002
0.001
0.000
Pald1+/+ (n=4)
Pald1-/- (n=4)
E
VEC
pY685 VEC
IsolectinB4
Pald1+/+
Pald1-/-
F
pY685 VEC area/ VEC area
1.0
0.8
0.6
0.4
0.2
0.0
Pald1+/+ (n=5)
Pald1-/- (n=7)

Figure EV5.
